# Delivery and late preterm birth

**DOI:** 10.1186/1824-7288-40-S2-A1

**Published:** 2014-10-09

**Authors:** Antonio Lanzone, Sergio Ferrazzani, Angela Botta

**Affiliations:** 1 Istituto di Clinica Ostetrica e Ginecologica, Università Cattolica del S. Cuore., Rome, Italy

## 

Delivery of infants who are physiologically mature and capable of successful transition to the extrauterine environment is an important priority for obstetric practitioners. During the past 15 years in the United States the percentage of infants born before 40 weeks’ gestation has dramatically increased and the percentage of infants born after 40 weeks’ gestation has decreased. In this shift several factors have been implicated: increased medical surveillance and interventions, increased multifetal pregnancies, maternal obesity (risk for preeclampsia, diabetes and other complications), maternal autonomy, route and timing of delivery.

Birth before fetal maturity contributes to short-term and long-term morbidity and mortality in late preterm (34^+0^ to 36^+6^ weeks’ gestation). Age stratified cohort studies confirms that adverse neonatal outcome decrease with increasing gestational age independent of delivery mode.

Because of the known morbidity and mortality associated with late preterm birth, iatrogenic delivery in this period has become a major concern. Preterm birth has been characterized as either “spontaneous” or “indicated.” For the most part, spontaneous late preterm births are difficult to avoid, whereas the term “indicated” implies that the delivery was necessary for maternal or fetal benefit. Gyamfi-Bannerman and colleagues found that 56.7% of late preterm non spontaneous deliveries were non-evidence based, concluding that more data were needed to justify many indications. A recent workshop by the Society for Maternal-Fetal Medicine developed consensus recommendations regarding the gestational age for delivery. These recommendations and those of the American College of Obstetricians and Gynecologists (ACOG) are based on the balance between maternal and newborn risks of early delivery with the risk of further continuation of pregnancy.

To decrease the mortality and morbidity associated with late preterm births, prevention is one of the key components. The ACOG does not recommend induced vaginal or planned cesarean delivery prior to 39 weeks gestation unless medically indicate. If elective induction is undertaken for nonmedical reasons, it should only take place if the preinduction assessment ensures the gestational age is at least 39 weeks.

In addition, further research is needed to refine the management of late preterm gestation (such as better identification of pregnancies that require early delivery for medical conditions):

• Assess the risk/benefit ratio for indications for late preterm delivery, such as more accurate estimation of fetal outcome in presence of maternal diseases.

• Identify management strategies to improve outcomes in late preterm infant (antenatal steroids)

• Improve the precision of determining gestational age.

